# Insights Into Sociodemographic Influences on Type 2 Diabetes Care and Opportunities for Digital Health Promotion in Port Harcourt, Nigeria: Quantitative Study

**DOI:** 10.2196/56756

**Published:** 2024-08-21

**Authors:** Oritsetimeyin Arueyingho, Jonah Sydney Aprioku, Paul Marshall, Aisling Ann O'Kane

**Affiliations:** 1 University of Bristol Bristol United Kingdom; 2 University of Port Harcourt Port Harcourt Nigeria

**Keywords:** type 2 diabetes, digital health, t2d in nigeria, technologies for diabetes, pharmaceutical care for t2d

## Abstract

**Background:**

A significant percentage of the Nigerian population has type 2 diabetes (T2D), and a notable portion of these patients also live with comorbidities. Despite its increasing prevalence in Nigeria due to factors such as poor eating and exercise habits, there are insufficient reliable data on its incidence in major cities such as Port Harcourt, as well as on the influence of sociodemographic factors on current self-care and collaborative T2D care approaches using technology. This, coupled with a significant lack of context-specific digital health interventions for T2D care, is our major motivation for the study.

**Objective:**

This study aims to (1) explore the sociodemographic profile of people with T2D and understand how it directly influences their care; (2) generate an accurate understanding of collaborative care practices, with a focus on nuances in the contextual provision of T2D care; and (3) identify opportunities for improving the adoption of digital health technologies based on the current understanding of technology use and T2D care.

**Methods:**

We designed questionnaires aligned with the study’s objectives to obtain quantitative data, using both WhatsApp (Meta Platforms, Inc) and in-person interactions. A social media campaign aimed at reaching a hard-to-reach audience facilitated questionnaire delivery via WhatsApp, also allowing us to explore its feasibility as a data collection tool. In parallel, we distributed surveys in person. We collected 110 responses in total: 83 (75.5%) from in-person distributions and 27 (24.5%) from the WhatsApp approach. Data analysis was conducted using descriptive and inferential statistical methods on SPSS Premium (version 29; IBM Corp) and JASP (version 0.16.4; University of Amsterdam) software. This dual approach ensured comprehensive data collection and analysis for our study.

**Results:**

Results were categorized into 3 groups to address our research objectives. We found that men with T2D were significantly older (mean 61 y), had higher household incomes, and generally held higher academic degrees compared to women (*P*=.03). No statistically significant relationship was found between gender and the frequency of hospital visits (*P*=.60) or pharmacy visits (*P*=.48), and cultural differences did not influence disease incidence. Regarding management approaches, 75.5% (83/110) relied on prescribed medications; 60% (66/110) on dietary modifications; and 35.5% (39/110) and 20% (22/110) on traditional medicines and spirituality, respectively. Most participants (82/110, 74.5%) were unfamiliar with diabetes care technologies, and 89.2% (98/110) of those using technology were only familiar with glucometers. Finally, participants preferred seeking health information in person (96/110, 87.3%) over digital means.

**Conclusions:**

By identifying the influence of sociodemographic factors on diabetes care and health or information seeking behaviors, we were able to identify context-specific opportunities for enhancing the adoption of digital health technologies.

## Introduction

### Background

Type 2 diabetes (T2D) is a metabolic condition characterized by elevated blood glucose levels and can lead to complications such as coronary heart disease and kidney failure [1]. Currently, approximately 90% of global diabetes cases are classified as type 2, affecting over 300 million individuals [2]. This represents about 8.3% of the world’s population. In Nigeria, a significant percentage of the population is affected by T2D. Similar to the rest of the world, the country is experiencing an increasing prevalence of the condition alongside other noncommunicable diseases [3]. According to the International Diabetes Federation, the estimated number of T2D cases in Nigeria is projected to increase from 3.6 million in 2021 to nearly 5 million by 2045 [4]. However, it is important to note that data regarding its prevalence in Nigeria appear inconsistent, with some research papers reporting rates of 5.7% [5], 4.3% [6], or even 11% [7]. In the South-South geopolitical zone of Nigeria, which includes Port Harcourt, some studies have indicated a prevalence of 9.8% and 6.8% of T2D in Port Harcourt itself, with varying values for other communities [8,9]. The increasing prevalence of T2D coexists with significant rates of communicable diseases, such as HIV [10,11]; infectious diseases, such as malaria [12,13]; and other noncommunicable diseases, such as cardiovascular and renal diseases [14]. The distinction between type 1 diabetes and T2D has also become blurred, and studies have shown that there has been no national consensus on diabetes prevalence since 1992 [15,16]. Given this disease burden and the lack of sufficient epidemiological data, T2D demands significant attention due to its association with distinct complications, many of which result in increased mortality.

Certain risk factors, such as obesity, genetics, sedentary lifestyle, and certain comorbidities, contribute to the incidence and onset of T2D. However, sociodemographic factors, including gender, age, ethnicity, and environmental influences, also play a significant role in increasing T2D risk and influencing the provision of care [17-20]. For instance, studies have shown that people with T2D residing in rural and remote regions face challenges with access to health care [21], such as lacking direct contact with a physician or registered pharmacist [22-24], dealing with unlicensed practitioners or complementary care providers [25], or being unable to manage referrals to secondary or tertiary health care institutions because of their physical inaccessibility [26,27]. Studies show that, globally, Black and Hispanic people face the highest risks of illness and death [28-30]. Another 20-year study found that Asian, Hispanic, and Black women have a much higher risk of T2D compared to White women, with increased BMI being especially harmful for Asian people [31]. Although T2D is mostly linked to wealthier and more developed areas, it is increasingly becoming a significant issue in developing countries like Nigeria [32-34]. This could result from cultural assimilation concerning food and lifestyle behaviors [35], potentially exacerbated by a lack of financial, educational, and infrastructural resources unique to the societal and community context.

### T2D Management

The management of the condition involves pharmacotherapy, which includes using hypoglycemic drugs (eg, sulfonylureas and meglitinides) and lifestyle modifications focusing on exercise and diet [36,37]. Digital interventions also play a role across different stages of T2D care, including diagnosis or prognosis with rapid test kits or machine-learning algorithms [38] and providing information, remote monitoring, and lifestyle modifications through mobile apps and websites [2,39,40]. Continuous glucose monitoring systems for blood sugar sensing and personalized medicine [41-43] are also critical components. Because T2D is a long-term condition that requires constant monitoring, its care involves self-management by people with the condition and collaborative care with both caregivers and health care practitioners in both formal and informal settings. However, we recognize that in certain contexts, such as Nigeria, there are limitations affecting the provision of care and the use of digital health technologies. There is also a dearth of contributions providing insight into the scope of T2D care and its intersection with sociodemographic and contextual factors influencing the use of digital health technologies. In light of these observations, we explore the Nigerian landscape by focusing on a notable developing city, Port Harcourt, to achieve the following objectives: (1) to explore the sociodemographic profile of people with T2D and understand how it directly influences their care; (2) to generate an accurate understanding of collaborative care practices, with a focus on nuances in the contextual provision of T2D care; and (3) to identify opportunities for improving the adoption of digital health technologies based on the current understanding of technology use and T2D care.

This was achieved through a quantitative study involving the collection of data from 110 people with T2D using traditional paper surveys and automated WhatsApp (Meta Platforms, Inc) surveys via Twilio (Twilio Inc), a cloud communications platform. We recognized the intersection between self-care and collaborative care practices, contextual influences, and technology use. We propose that insights from our study will contribute to the body of work on T2D incidence and prevalence in Port Harcourt, Nigeria, as well as provide guidance on the design of context-specific digital interventions for T2D care.

## Methods

To uncover the influence of contextual factors on T2D care, the current state of technology use for T2D care, and potential design opportunities to improve self and collaborative T2D care, we obtained 110 responses from questionnaires distributed both on paper and via an automated WhatsApp system.

### Positionality

The lead researcher, who is also the first author, has a diverse background as a pharmacist, health care manager, and digital health scholar. Having resided, studied, and practiced in Port Harcourt, Nigeria, for >2 decades, their motivation for undertaking this study is deeply rooted in personal experiences acquired while working as both a hospital and community pharmacist. During this period, they had the opportunity to interact with a wide range of patients, which led to a profound realization of the critical role that cultural diversity plays in the delivery of pharmaceutical care.

The second and third authors, specializing in human-computer interaction, have a collective research focus on the use of health and wellness technologies within various contexts. They bring a valuable interdisciplinary perspective to the study. The fourth author, based in Nigeria, is a pharmacist and academic with a wealth of expertise in the field of T2D research. His work has primarily concentrated on the development of context-specific public health interventions, and he also demonstrates a keen interest in exploring the potential of digital health interventions.

### Study Design and Setting

This quantitative cross-sectional study, which was conducted from February through April 2023, involved respondents who were contacted using random and snowball sampling techniques.

### Ethical Considerations

The study was made possible by ethics approval from the research ethics committee of the University of Port Harcourt, Nigeria (UPH/CEREMAD/REC/MM85/044). Participants were required to provide informed consent twice, the first being when they signed and filled in the expression of interest form on the web, and the second when they began to answer the questionnaire. Participants were not financially compensated and personal identifiable data such as names were anonymized.

### Study Site

Nigeria is currently the most populous country in Africa, with an estimated 223 million people and >371 tribes [44]. This population is expected to grow steadily in the coming years, which will place additional demands on the health care system. Our study was conducted in Port Harcourt, a major developing city in Nigeria with a significant prevalence of T2D [45]. It has a rich colonial history and is a vibrant melting pot of diverse ethnicities, with numerous languages spoken.

### Study Population

On the basis of the objectives of this study, the intended questionnaire respondents were people with T2D residing in Port Harcourt, Nigeria. Eligibility criteria required participants to have been diagnosed with T2D for >6 months, be aged >18 years old, and be capable of providing informed consent. We obtained a total of 110 questionnaire responses, comprising 27 valid responses from the automated WhatsApp questionnaires and 83 responses from the questionnaires distributed in person.

### Study Instruments

Data were obtained using paper questionnaires and WhatsApp, automated with Twilio. The data were analyzed using descriptive and inferential statistics with the SPSS Premium (version 29; IBM Corp) and JASP (version 0.16.4; University of Amsterdam) software.

### WhatsApp for Data Collection

WhatsApp is the most pervasive instant messaging platform in Nigeria, with at least 40% of its population being active users [46]. While quantitative data collection in contexts such as that in Nigeria mostly involves distributing surveys either electronically through Microsoft Forms (Microsoft Corp) or other avenues or in person through paper, the collection of quantitative data using WhatsApp remains underexplored. WhatsApp offers a cost-effective approach for both respondents and researchers. It is user-friendly for people already accustomed to WhatsApp chat functions and ensures sustained participation among mobile populations, as users can retain their WhatsApp number even when changing SIM cards or phones. To recruit WhatsApp respondents, we used a different approach (discussed subsequently) compared to traditional survey distribution methods. This allowed us to reach a hard-to-reach audience, including participants who might otherwise have been unreachable in person. This validates WhatsApp’s utility and adds to its list of advantages. However, researchers lacking technical skills or familiarity with web-based recruitment methods may perceive this approach as a limitation.

**Figure 1:**
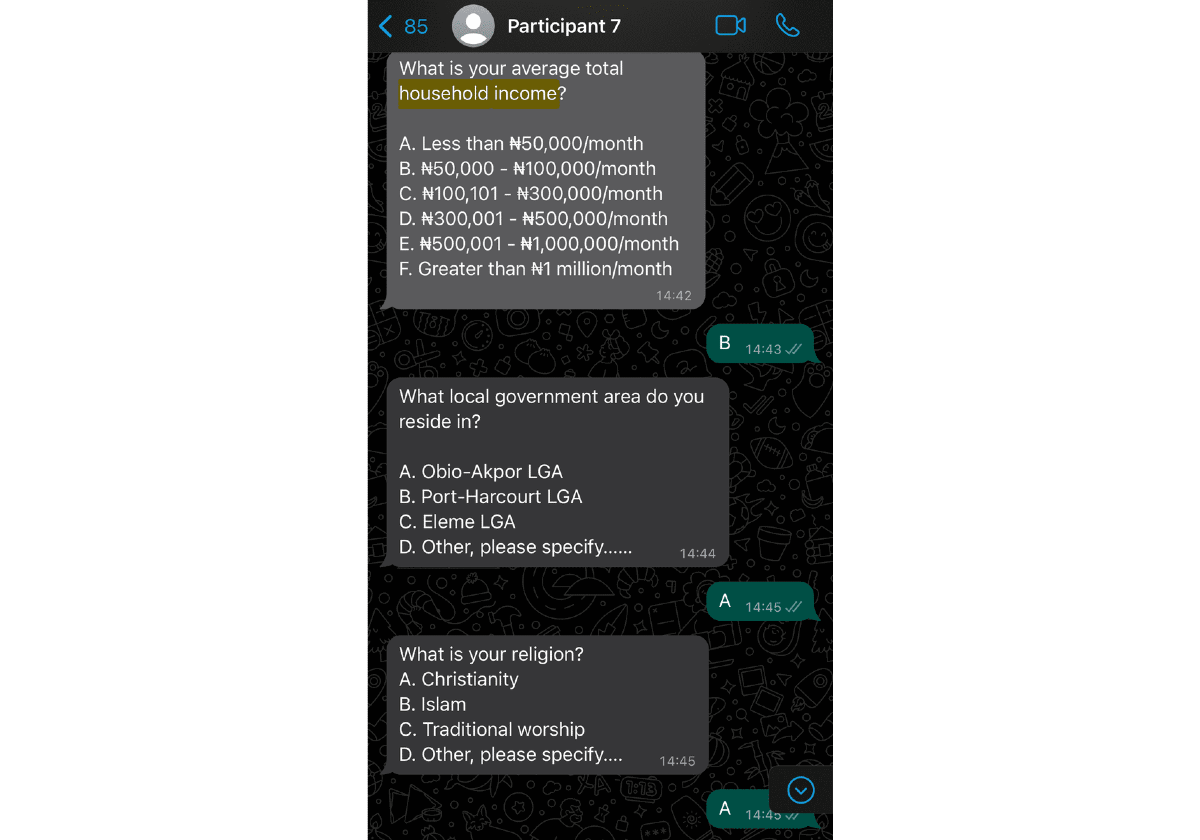
Data collection on WhatsApp (Meta Platforms, Inc) enabled by Twilio (Twilio Inc).

We obtained quantitative data by automating WhatsApp using Twilio. This approach was a replication of the method used by Fei et al [47] in their work on automated WhatsApp questionnaires in panel surveys. The phone numbers of people who expressed interest via the form we disseminated on social media were securely stored in a password-protected Google Sheet (Google LLC). Subsequently, we created a WhatsApp questionnaire using Twilio’s studio dashboard for questionnaire flow. Google Apps Scripts were used for automating questions, allowing participants to respond within the WhatsApp environment. In the Twilio platform, each response from the participants led to the transfer of the response to the password-protected Google Sheet used. On the participants’ part, they were instructed to respond using single letters to indicate their chosen answers for each question. Subsequently, this action triggered the presentation of the next question, which also necessitated a single-letter response (Figure 1). This process continued until the questionnaire was completed, and every respondent who expressed interest filled out the WhatsApp questionnaire.

### Questionnaire Design

An expert panel consisting of the first author, 2 human-computer interaction experts, and an academic pharmacist residing in Port Harcourt designed the questionnaires in accordance with the research objectives and outcomes derived from preorganized synchronous WhatsApp interviews. To provide context, a total of 36 individuals participated in the preceding WhatsApp interviews, including 15 (42%) people with T2D, 9 (25%) community pharmacists, and 12 (33%) caregivers. Through these interviews, we gathered valuable insights regarding the scope of T2D care in Port Harcourt, the influence of health care providers on community T2D care, potential design opportunities for T2D care, and the role of instant messengers and social media in data collection and T2D care. These insights informed the iterations made to the questionnaire during its design phase. The questionnaires were specifically targeted at people with T2D to obtain statistical information that would answer questions regarding demographics, disease prevalence and coexistence with other morbidities, the role of technology, and how technology is adopted and integrated into their care.

We used a mixed-question format in the design of the questionnaire, incorporating both open-ended questions that required written responses, such as “What approach best describes how you manage your condition?” and closed-ended questions with predefined options, such as “Do you use any form of technology in the management of your condition?” Following the completion of the questionnaire design, we conducted a pilot study involving 6 participants. Among them, 2 (33%) participants resided in the United Kingdom, while the remaining 4 (67%) were based in Nigeria. The 2 pilot participants from the United Kingdom came from diverse backgrounds, including psychology and statistics. Their feedback was invaluable in assessing the questionnaire’s readability and statistical validity. The individuals who participated in the pilot study from Nigeria were primarily pharmacists and academics. They offered insights into the sociocultural relevance of the questions and provided feedback on the questionnaire’s reproducibility and comprehensibility. Following the conclusion of the pilot study, we made iterative improvements to the questionnaires, and the final versions were either printed or manually transferred to Twilio’s dashboard for data collection.

### Sample Size Calculation

In the absence of consistent epidemiological data on the prevalence of T2D in Port Harcourt and Rivers state, we used a standard formula to estimate the required sample size based on the established prevalence rate of T2D in Nigeria [9]. The formula required the input of a constant (SD set at 1.96), corresponding to a 95% CI; the estimated proportion of the population with T2D, which was calculated to be 0.036 (3.6% prevalence rate); and a margin of error of 0.05. The ideal sample size for this study was determined to be 55.31.

*N* = [*Z*^2^ × P (1 – *p*)] / *E*^2^
**(1)**

Using the formula for design effect to calculate anticipated attrition of 10%, we obtained 61 as the recommended number of responses in the study. Since this systematic sampling technique ensures the appropriate sample size is obtained, but not necessarily an equitable representation of the sample, and due to inconsistencies with data on the prevalence rate of T2D in Port Harcourt or Rivers state, we aimed to double the initially recommended sample size (*N*×2=110) to achieve an adequate representation of people with T2D.

1 / (1 – adjusted factor) × estimated sample size = ideal number of participants **(2)**

(1 / [1 – 0.1]) × 55 = 61 **(3)**

### Data Collection

Participant recruitment was carried out through a combination of random and snowball sampling techniques. For the WhatsApp questionnaires, an expression-of-interest form was created and distributed on social media. An advertisement designed to run for 2 weeks at US $2 per day was created on a designated Facebook (Meta Platforms, Inc) page with no followers for the questionnaire. It featured an image depicting the disease condition in people of color and a concise title: “Have Type 2 Diabetes? Sign up; let’s take action.” A summary of the study was included in the description section, and the advertisement was targeted at people living in Port Harcourt. Although the advertisement reached 3127 individuals and received approximately 46 likes, 10 shares, 2 comments, and 107 link clicks, we excluded 23 invalid responses and obtained only 27 valid responses, as the form was designed to filter out ineligible participants. Out of the 23 invalid responses, 15 (65%) were excluded because the respondents did not have T2D, and 8 (35%) were excluded because they did not provide contact information. Selected participants were contacted using the email addresses or WhatsApp phone numbers they provided on the forms.

Conversely, the distribution of physical questionnaires was facilitated through referrals from pharmacists; nongovernmental organizations, such as The Diabetes Care Network (a Port Harcourt–based nongovernmental organization focused on the provision of diabetes-related health education and digital health care); and random distributions in open markets, academic institutions, and churches. Although we distributed hundreds of questionnaires, we obtained a total number of 87 questionnaires, out of which 83 (95%) were valid. Questionnaires were invalidated for reasons such as being incomplete, ineligible, or suspicious.

### Data Analysis

#### Overview

First, the first author exported the WhatsApp questionnaires as a zip file without any media attachments. The transcripts in the file, in .tex format, were compared to the Google Sheets responses for cleaning and editing. Thereafter, data in the traditional questionnaires were manually transferred to a Microsoft Excel sheet where they were cleaned. Both quantitative data sets were merged as a CSV file before being exported to the SPSS or JASP software for statistical analysis.

#### Statistical Analysis

Characteristics of people with T2D are reported as mean and SD for continuous variables such as age and duration of stay in Port Harcourt, while they are reported as percentages for categorical variables. These analyses were performed using univariate comparisons. To compare differences in the sociodemographic variables of women and men, we used a chi-square test for categorical variables, such as education, household income, and occupation, and an independent samples 1-tailed *t* test for continuous variables, such as age and duration of stay in Port Harcourt. To test the relationship between gender and how the condition is managed, either via hospital visits or via pharmaceutical care, we used a logistic regression model. To compare differences in sociodemographic variables using the chi-square test, we organized our data into rows and columns. The data were separated into 2 groups based on gender, and we encoded men and women as 0 and 1, respectively. Subsequently, we assigned numerical values to represent the options for each sociodemographic-related question. Finally, we conducted the relevant analysis using the SPSS software. A similar approach was used for cleaning and organizing data in the independent samples 1-tailed *t* test, with the difference being the use of continuous variables this time. For the logistic regression model, we organized our data sets into columns representing relevant categorical variables. Thereafter, we encode the responses into numerical values; for example, for the question indicating our independent variable “What approach describes how you manage the condition?” we code option A, which is prescribed medicines, as 0. Other options are coded in an ascending numerical order. For dichotomous responses, such as that observed for the question “Do you have a mobile phone?” we encoded the options yes and no as 0 and 1, respectively. We selected gender as the dependent variable that we want to predict, in comparison with other independent variables, such as the frequency of hospital visits and pharmacy visits, which were categorical variables in the questionnaire. We used the length of the period with T2D and reasons for visiting either the hospital or pharmacy as covariate factors. The data were cleaned and saved in a CSV file, which was analyzed in the JASP software.

## Results

### Summary of Key Findings

The key findings of this study have been summarized in Textbox 1.

**Key findings.**

**Findings**

1. Cleaning the data resulted in an equal number of men and women in the study. However, people identifying as men were significantly older and had a higher average household income compared to those identifying as women. They also mostly accounted for those recently diagnosed with type 2 diabetes (T2D) and those living with the condition for more than >5 years (Table 1).
2. More than 92.7% (102/110) of people with T2D sampled in this study had tertiary education, resulting in a high literacy rate among our questionnaire respondents. This trend may be attributed to the venues where participant recruitment occurred, referrals from community pharmacists, potential bias in questionnaire distribution, and the assumption that mostly literate individuals would express interest in participating in the recruitment process on the web.
3. Most questionnaire respondents had a hybrid approach to managing their condition, combining prescribed medications with traditional medicines or other lifestyle modifications. Very few respondents admitted to using prescribed medications alone (Table 2).
4. Most questionnaire respondents who admitted to using technology to manage their condition claimed that they were using glucometers. Glucometers provide valuable information that helps people make informed decisions about their diabetes management; however, they are not necessarily interventions for actively managing symptoms, complications, or any indication of the disease (Table 2).
5. Physical contact was the most preferable option for questionnaire respondents for managing the condition and accessing related health information.
6. WhatsApp and Facebook were the most popular instant messaging and social media platforms, respectively, among questionnaire respondents. However, communication with pharmacists over these platforms rarely occurred, and when it did, it was mostly in the form of chats.

**Table 1:** Sociodemographic profile of men and women with type 2 diabetes in Port Harcourt (N=110).

Variable		Men (n=55)	Women (n=55)	*P* value	
Age (y), mean (SD)		61 (1.15)	48 (2.16)	.03	
**Level of education, n (%)**					.04
	Secondary school	1 (2)	1 (2)		
	College or polytechnic	1 (2)	2 (4)		
	University (bachelor’s degree [BSc^a^])	35 (64)	40 (73)		
	Master’s degree (MSc^b^)	16 (29)	11 (20)		
	Doctorate	2 (4)	1 (2)		
**Household income per month ( 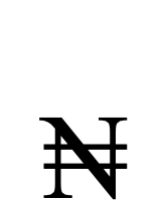 ), n (%)**					.58
	<50,000 (US $35.7)	1 (2)	3 (5)		
	50,000 (US $35.7) to 100,000 (US $71.4)	15 (27)	19 (35)		
	100,000 (US $71.4) to 300,000 (US $214.3)	31 (56)	29 (53)		
	300,000 (US $214.3) to 500,000 (US $357.1)	6 (11)	3 (5)		
	>500,000 (US $357.1)	2 (4)	1 (2)		
**Occupation, n (%)**					.09
	Student	1 (2)	2 (4)		
	Self-employed	19 (35)	22 (40)		
	Professional	21 (38)	15 (27)		
	Manager or executive	5 (9)	1 (2)		
	Housewife or husband	1 (2)	10 (18)		
	Religious leader	4 (7)	2 (4)		
	Unemployed	1 (2)	1 (2)		
	Retired	3 (5)	2 (4)		
**Mobile phone type, n (%)**					.01
	Android	45 (82)	34 (62)		
	iPhone	9 (16)	21 (38)		

^a^BSc: Bachelor of Science.

^b^MSc: Master of Science.

**Table 2:** Current T2D^a^ self-care practices, including the use of digital interventions (N=110).

		Men (n=55), n (%)	Women (n=55), n (%)
**How long have you been diagnosed with T2D?**			
	6 months-1 year	20 (36)	15 (27)
	1-5 years	27 (49)	37 (67)
	>5 years	8 (15)	3 (5)
**What approach best describes how you actively manage the condition (select more than one if necessary)?**			
	Prescribed medications	38 (69)	45 (82)
	Traditional medicines	23 (42)	16 (29)
	Dietary changes	30 (55)	36 (65)
	Exercise	18 (33)	27 (49)
	Spirituality (eg, prayers)	9 (16)	13 (24)
	Technology	4 (7)	2 (4)
**Do you use any form of technology in the management of your condition?**			
	Yes	12 (22)	16 (29)
	No	35 (64)	36 (65)
	Unsure	8 (15)	3 (5)
**If yes, what best describes the form of technology that you use to manage your condition (select more than one if necessary)?**			
	Glucometer (the device to check your blood sugar levels)	51 (93)	47 (85)
	Websites (eg, offers advice on self-care)	1 (2)	3 (5)
	Mobile apps (eg, helps track blood sugar and activity)	3 (5)	5 (9)
**If no, what best explains why you do not use any of the above?**			
	Too expensive	12 (22)	14 (25)
	I do not know about them	12 (22)	4 (7)
	I prefer physical contact	27 (49)	35 (64)
	It is against my religious beliefs	4 (7)	2 (4)
**If yes, what best explains why you use any of the above (select all that apply)?**			
	It is affordable	40 (73)	29 (53)
	It is general practice	37 (67)	31 (56)
	No reason, I do not think about technology much	4 (7)	5 (9)
	I was convinced by someone	8 (15)	11 (20)

^a^T2D: type 2 diabetes.

### Sociodemographic Characteristics of People With T2D

#### Positive Relationship Among T2D Prevalence, Comorbidities, and Age

An equal number of people who identified as men or women actively engaged with the questionnaires (Table 1). The mean age of people who identified as men was 61 (SD 1.15) years, while that of those who identified as women was 48 (SD 2.16) years. It was observed that a substantial percentage of women with T2D (44/55, 80%) ranged between the ages of 40 and 55 years, compared to men with T2D who mostly ranged between 45 and 65 years. A total of 58.2% (64/110) of people with T2D reported that they had been living with the condition for >1 but <5 years. Women had the highest prevalence within this time frame, accounting for 33.6% (37/110) of people living with T2D. However, men accounted for over 55% (11/20) of those recently diagnosed with T2D, that is, within 6 months to a year. While we cannot out rightly state that there is a positive relationship between T2D prevalence and gender because we did not consider confounding factors, we observed that there was a relationship between an increase in age and the prevalence of the condition.

#### High Household Income Linked to Education and Occupation

Less than 1.8% (2/110) of the questionnaire respondents had no postsecondary school education. However, most respondents possessed some level of education, with 68.1% (75/110) holding bachelor’s degrees, 24.5% (27/110) holding master’s degrees, and 2.7% (3/110) having doctorates (Table 1). An additional 2.7% (3/110) of the population held various academic certificates, including diplomas and the West African Senior Secondary School Certificate. While no positive relationship was observed between education and the prevalence of the disease, a positive relationship was noted among education, occupation, household income, and the incidence of the disease. It was observed that people with T2D who held Bachelor of Science and Master of Science degrees were mostly self-employed (41/110, 37.3%) or professionals (35/110, 31.8%), such as engineers and lawyers. They predominantly earned between 
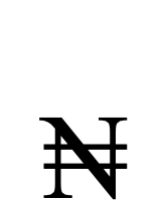
100,000 (US $ 71.4) and 
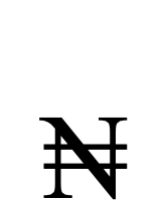
300,000 (US $214.3). Finally, most people with T2D who were aged >55 years held managerial positions or served as religious leaders and claimed to earn between 
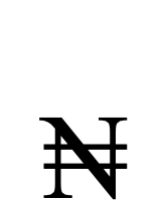
300,000 and 
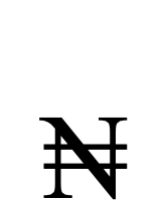
500,000 (US $214.3-$ 357 at the time of writing).

#### State of Origin (Cultural Differences) Does Not Influence T2D Prevalence

Participants were asked to indicate their state of origin and how long they had resided in Port Harcourt. A total of 15 states were identified, with Rivers (24/110, 21.8%), Anambra (17/110, 15.5%), and Edo (12/110, 10.9%) states accounting for the top 3, while Kwara (1/110, 0.9%), Plateau (1/110, 0.9%), and Ondo (1/110, 0.9%) states represented the bottom 3. While 32.7% (36/110) of people with T2D noted that they had resided in Port Harcourt for 1 to 10 years, most of them from Anambra, Imo, and Delta claimed to have lived in Port Harcourt for 6 to 10 years. People with T2D who had resided in Port Harcourt for >10 years were mostly from Rivers, Bayelsa, and Abia. As we did not ask about their state of birth, we could not determine whether their residence in Port Harcourt was a result of migration or due to birth. Also, we did not observe any statistical relationship between the state of origin of respondents with T2D and T2D prevalence.

### Collaborative T2D Care: Self Practices, Caregiving, and Pharmaceutical Care

#### Varied Self-Care Practices Influencing Hospital Visits

Most people with T2D (64/110, 58.2%) stated that they had been diagnosed with T2D for 1 to 5 years), while 29.1% (32/110) were diagnosed within the past year (Multimedia Appendix 1). When asked to signify what approach best describes how they manage their condition, most people with T2D noted that they depended on prescribed medications (83/110, 75.5%) and dietary modifications (66/110, 60%). The use of traditional medicines (39/110, 35.5%) and exploration of spirituality (22/110, 20%) in the management of T2D was not left out, as it was observed that they actively combined these approaches with dietary changes and prescribed medications.

We observed no statistically significant relationship between gender and the frequency of hospital (*P*=.60) or pharmacy visits (*P*=.48). However, most people with T2D (69/110, 62.7%), including those who depended on prescribed medications claimed to visit the hospital twice a year for care related to their condition. Surprisingly, 7.3% (8/110) admitted to never visiting the hospital, and it was observed that they selected traditional medicines and spirituality as their preferred approach for T2D care. Furthermore, 62.7% (69/110) of people with T2D reported that they visited the hospital only when they had complications, and this accounted for 80% (88/110) of the individuals who claimed to visit the hospital twice a year. In total, 20% (22/110) of questionnaire respondents implied that they visited hospitals, when necessary, in the blank options provided in the questionnaire. However, 15.5% (17/110) of them still indicated that they visited the hospital twice a year. Furthermore, 28.2% (31/110) of questionnaire respondents noted that they visited the hospital majorly for medication refills; however, 70.9% (78/110) of them said that they visited the hospital every 3 months. While university teaching hospitals were the most frequently visited (40/110, 36.4%) and private hospitals were the least frequently visited (3/110, 2.7%), most people with T2D who noted that they visited the hospital majorly for blood glucose and diabetes checkup selected primary health centers as their most frequently visited institutions. We did not ask questions to uncover the reasons behind their hospital choice, but we did observe that the majority of the respondents who claimed to visit the hospital twice a year admitted to visiting the pharmacy every month.

#### No Relationship Between T2D Prevalence and Caregiving or Pharmaceutical Care

Each person with T2D noted that they visited community pharmacies, albeit with varying frequencies (Multimedia Appendix 2). Most respondents (95/110, 86.4%) indicated that they visited community pharmacies every month, and approximately 1.8% (2/110) noted that they visited community pharmacies twice a year. We did not uncover the factors influencing the frequency of their visits to community pharmacies in the questionnaire; however, we determined the reasons why they chose to visit community pharmacies. They mostly visited community pharmacies for medication refills (74/110, 67.3%) and for disease complications (50/110, 45.5%). We observed that only 80.9% (89/110) of questionnaire respondents admitted to purchasing medications from community pharmacies, including those who indicated that they visited community pharmacies quite frequently. Patent medicine stores (58/110, 52.7%) and health multilevel marketing companies (17/110, 15.5%) accounted for a significant proportion of where T2D medications were routinely obtained from. Communication with pharmacists was mostly in person (98/110, 89.1%), and for people with T2D who engaged with their pharmacists on social media or instant messengers, WhatsApp was the most popularly used platform (85/110, 77.3%). Furthermore, 35.5% (39/110) of people with T2D indicated that they had a caregiver assisting with their disease condition, while 37.3% (41/110) noted that they occasionally had a caregiver. In addition, 69.1% (76/110) of questionnaire respondents indicated that their children were their primary caregivers. Respondents who indicated that they had active caregivers and that they were their children were mostly men (65/110, 59.1%). Women who selected children as their primary caregivers were mostly aged >50 years. We observed that most respondents who indicated that they visited the hospital every 3 months had their children as primary caregivers, were aged >55 years, and earned above 
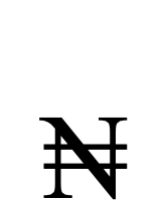
300,000 (US $214.2). However, we could not positively or negatively correlate this observation with the prevalence of T2D.

#### Management of T2D and Comorbidities Supported by Formal Education

We discovered that people identifying as men generally held higher academic degrees than women (*P*=.03). However, it is noteworthy that women tended to have more bachelor’s degrees than men. This difference might be attributed to the significant age gap between both genders, the nature of participant recruitment, or other sociocultural factors that were not considered. A positive relationship between the level of formal education and approach for the management of T2D was observed. Most people who had a bachelor’s degree indicated that they relied on prescribed medications to manage their condition. There was an equal distribution between those who visited the hospital twice a year and those who had scheduled routine visits for medication refills, often associated with comorbidities. We noted the presence of comorbidities such as hypertension (79/110, 71.8% of questionnaire respondents), cardiac related diseases (35/110, 31.8%), kidney diseases (19/110, 17.3%), and cancer (3/110, 2.7%). Finally, individuals with a doctorate degree who were all aged >55 years reported making routine visits to both the hospital and the pharmacy for the management of T2D and coexisting conditions. They were either retired or held executive positions, resulting in higher household incomes. These factors may have contributed to their health-seeking behaviors.

### Digital Health Approaches for T2D Care

#### Most People With T2D Were Familiar Only With Glucometers

Our findings suggest that 64.5% (71/110) of questionnaire respondents did not use any forms of technology to manage their T2D, and 10% (11/110) of questionnaire respondents were unsure about whether they used technology to manage their disease condition. We observed that most respondents who indicated that they use technology to manage their condition had mobile phones with access to the internet. The form of technology being referenced by questionnaire respondents was mostly a glucometer (98/110, 89.2%), as no respondent indicated something different in the blank spaces. The lack of glucometer use was significantly impacted by varying factors, including cost (26/110, 23.6%), ignorance about use (16/110, 14.5%), preference for physical contact (66/110, 60%), and religious preferences (2/110, 1.8%). Finally, we observed that some respondents who selected mobile apps and social media as their source of information did not consider these approaches as forms of technology for managing their disease condition.

#### Mobile Phones Relevant in T2D Care

Of the questionnaire respondents, 99.9% (109/110) indicated ownership of a mobile phone. However, most users reported having Android phones (79/110, 71.8%), among which 31.8% (35/110) had steady access to the internet, and these users were predominantly men. Unfortunately, we did not observe any significant relationship among the type of mobile phone used, household income, and occupation. Therefore, there is no clear explanation for why iPhones (which are typically more expensive) were owned majorly by women. In addition, considering that we did not probe further to inquire on the model and brand of the respondents’ phones and that we did not observe a significant relationship among mobile phone type, household income, and occupation, we cannot infer that Android phones were significantly cheaper, that respondents earned enough to purchase them, or that they were purchased with managing a health condition in mind.

#### WhatsApp Facilitates Communication, While Facebook Acts as an Information Source

The findings from the questionnaire revealed that both men and women with T2D regularly used WhatsApp for communication (Table 3); however, the focus on its use for T2D care was unexplored. While WhatsApp emerged as the most popular instant messenger, it was not frequently used for communication with pharmacists. According to our findings, men reportedly contacted pharmacists more frequently on WhatsApp compared to women, and they primarily did so through regular chats. By contrast, our questionnaire data showed that women were more likely to collaborate on WhatsApp through conference calls or group chats with family or health care practitioners for health-related reasons. Furthermore, Facebook was reported as the most popular social media network, especially among men with T2D. However, concerning T2D care, few respondents acknowledged its use. An interesting observation was the selection of *word-of-mouth* (62/110, 56.4%) as the most accessible means for obtaining T2D-related health information.

**Table 3:** Communication and information seeking modalities (N=110).

		Men (n=55)	Women (n=55)
**What instant messenger do you use regularly?**			
	WhatsApp	42 (76)	42 (76)
	Telegram	11 (20)	11 (20)
	Facebook Messenger	2 (4)	2 (4)
	Other	—^a^	—
**What social media network do you use regularly?**			
	Facebook	51 (93)	51 (93)
	Instagram	1 (2)	1 (2)
	Twitter	3 (5)	3 (5)
	TikTok	—	—
	Other	—	—
**Do you communicate with your pharmacist using WhatsApp?**			
	Yes	22 (40)	22 (40)
	No	33 (60)	33 (60)
**If yes, select the way you use WhatsApp to communicate (select more than one if necessary; n=22)**			
	Video call	1 (4)	1 (4)
	Web-based call	4 (18)	4 (18)
	Conference call with my family or a physician	—	1 (4)
	Group chats with family, a pharmacist, or physician	—	3 (14)
	Regular chats (texts)	22 (100)	17 (77)
**How do you assess health information related to managing type 2 diabetes (select more than one if necessary)?**			
	Mobile apps	4 (7)	6 (11)
	Social media	12 (22)	17 (31)
	Radio	—	—
	Television	2 (4)	—
	Newspapers	1 (2)	—
	Web-based courses	8 (15)	12 (22)
	Word of mouth	49 (89)	47 (85)
	Other (please specify)	—	—

^a^Not applicable.

## Discussion

### Principal Findings

Our findings align with global trends suggesting an increasing prevalence of T2D with advancing age, likely influenced by hormonal and lifestyle factors [48,49]. Older individuals tend to have higher educational attainment and increased household income, although this correlation may not always hold true in rural areas. Studies conducted in Nigeria indicate that these factors significantly influence health-seeking behaviors [50,51], including preferences for hospital visits, community pharmacies, or traditional medicine practices. Moreover, our observations reveal that cultural differences based on ethnicity or state of origin do not significantly impact the scope of T2D care, emphasizing the need for nationwide health policies and interventions tailored to address regional disparities. These policies should promote holistic approaches that consider contextual factors influencing health-seeking behaviors [52]. Findings from our study also highlight several opportunities for context-specific health care solutions, such as implementing health literacy programs, developing culturally appropriate diabetes management guidelines, and advocating for policy reforms to ensure equitable health care provision across diverse sociodemographic groups.

Our study indicates that a significant portion (71/110, 64.5%) of respondents do not use any form of technology for managing their T2D, with a notable proportion citing cost, ignorance about technology use, preference for physical contact, and religious preferences as barriers to adopting technologies, such as glucometers. This finding underscores the need for targeted educational campaigns to address misconceptions and enhance awareness about the benefits of technological tools, for example, large language models, such as Nigerian-owned “Awarri” in diabetes management [53-57]. This could be a significant opportunity for improvement by the Nigeria Digital in Health Initiative focused on transforming the digital health care landscape of Nigeria. Furthermore, the prevalence of mobile phone ownership (109/110, 99.9%) among respondents, predominantly Android phones with internet access, suggests an opportunity to leverage mobile platforms for delivering health information and interventions. Our study, similar to others [6,58,59], reveals that “*word-of-mouth*” is the most accessible source of T2D-related health information for respondents. This preference suggests a reliance on interpersonal networks and community-based information dissemination rather than formal health care channels. Integrating digital health practices and locally relevant health messaging into community networks could enhance the dissemination of digital health information and improve health and technology literacy among person with T2D.

### Context-Specific Opportunities for Enhancing the Adoption of Digital Health Technologies

Key findings from this study indicate the need for technological interventions to support T2D selfcare and collaborative care. They are relevant to not only people living with T2D in Port Harcourt but also those in Nigeria as a whole. Opportunities for improvement are summarized in Textbox 2.

**Improvement opportunities.**

**Opportunities**

1. The notable percentage of educated individuals with type 2 diabetes (T2D) represents a promising opportunity to facilitate data-driven approaches, digital health promotion, and co-design activities for a suitable T2D intervention. These can be leveraged and designed to suit the context, as general interactive and personalized resources can be understood by this audience. However, because most respondents were aged >50 years (as some studies have shown that digital literacy is lower in older demographics), they may have limited technological proficiency and could benefit from guidance, assistance, and support [60-62]. Bridging the digital divide by providing sustainable policies and digital health education could go a long way in promoting the use of digital health technologies as well as give users a positive perception about them.
2. Although most questionnaire respondents earned considerably above the average income by Nigerian standards, it may not be a generalizable experience. Considering the indigenous design and deployment of interventions would not only minimize the influx of Western and noncontextual interventions to this populace but also make them more affordable and accessible to Nigerian residents. This is because certain costs, such as incurred taxes, importation fees, distribution, and mark-up fees, would be avoided. In addition, fluctuations in the exchange rate would have minimal influence on domestic prices. This highlights a significant opportunity in the Nigerian market for indigenously designed interventions.
3. Most respondents managed their health based on information that they had learned from social media (presumably Facebook; 83/110, 75.5% general use), their physicians, family members, or other health care providers within their reach. Therefore, relevant interventions should be designed to be integrated with existing routine self-care and collaborative care practices and accessible technologies such as instant messengers and other mobile apps [40]. Given that 61.8% (68/110) of respondents cited “general practices” as a reason for using technology (mostly glucometers), there is a significant opportunity to encourage technology adoption and minimize resistance by integrating these efforts: creating awareness of digital health interventions, improving digital health policies, engaging with end users through design activities, and deploying Nigerian-owned products in general practices.
4. The preference for in-person interactions may be due to a myriad of factors, including cultural influences; expenses; misinformation; and a lack of exposure, awareness, and knowledge about digital interventions. Creating awareness about digital interventions during in-person interactions by health care practitioners; slowly integrating them into health care structures; and leveraging artificial intelligence, virtual reality, and other techniques might be a strategic way to encourage the use and adoption of digital health interventions in this research context [63,64].

### Limitations

The limitations of this study are follows. First, we did not inquire about the number of people per household, nor did we consider the purchasing power parity of the Naira to estimate the adequacy of their household income. Our study did not account for the presence of other financially demanding factors or the influx of income through secondary activities. Such considerations would, in turn, have provided a more accurate perception of the affordability of relevant technological interventions. Second, the questionnaire did not include questions about suitable digital alternatives other than WhatsApp for specifically communicating with pharmacists. In addition, there were no questions related to understanding their precise digital health needs. We omitted these questions intentionally to gather initial insights from the questionnaire, which would then inform subsequent interviews involving more in-depth probing. Third, our approach to disseminating the questionnaires may have led to an unfair representation of people with T2D in Port Harcourt, resulting in a high percentage of educated individuals earning above the country’s average. In-person questionnaires were likely administered to individuals who appeared literate and were easily accessible. The primary researcher received fewer responses from dissemination campaigns in open markets compared to dissemination campaigns in outpatient primary health diabetes centers, academic institutions, and churches. In addition, it is anticipated that individuals with limited education or restricted access to mobile phones, social media, or the internet would be less likely to express interest in the study on the internet. Fourth, unlike paper questionnaires that can be shared in person, respondents for automated WhatsApp questionnaires need to be recruited beforehand. While this offers the advantage of ensuring that potential participants understand the study and are genuinely interested in participating, it can also be a potentially stressful and expensive process. Researchers would need to learn about the automation of WhatsApp using suitable cloud communication platforms such as Twilio or infobip and run targeted social media campaigns on designated pages. Considering that we spent approximately US $28 for our campaign and obtained only 27 valid responses, we assume that obtaining a significant amount of data would be costly and potentially stressful using this approach.

### Conclusions

On the basis of the scarcity of research addressing sociodemographic factors influencing the use of technology for T2D care in our research context, this study highlights significant opportunities for fostering self-care and collaborative care using technology. This study unveils key relationships between contextual and epidemiological factors of the condition and how they influence health-seeking behaviors and care practices, which, in turn, would inform design decisions. We can assert that people with T2D residing in the city of Port Harcourt are receptive to technological interventions for managing their condition. Although the specific nature of this intervention remains uncertain, we are confident that, with the right approach, issues related to use, adoption, compliance, and referral will not pose significant challenges. We advocate for the replication of this study in similar Global South contexts, incorporating any overlooked questions, exploring alternative platforms to Twilio, and refining sampling or recruitment practices.

## Appendix

Multimedia Appendix 1 Logistic regression model showing the relationship between gender and the categorical frequency of hospital visits.

Multimedia Appendix 2 Logistic regression model showing the relationship between gender and the categorical frequency of pharmacy visits.

## Abbreviations

T2D: type 2 diabetes
