# Supplementary figures and images for: Insights Into Sociodemographic Influences on Type 2 Diabetes Care and Opportunities for Digital Health Promotion in Port Harcourt, Nigeria: Quantitative Study
Source: JMIR Diabetes. 2024 Aug 21;9:e56756. doi: 10.2196/56756 (PMC11375378; doi:10.2196/56756)

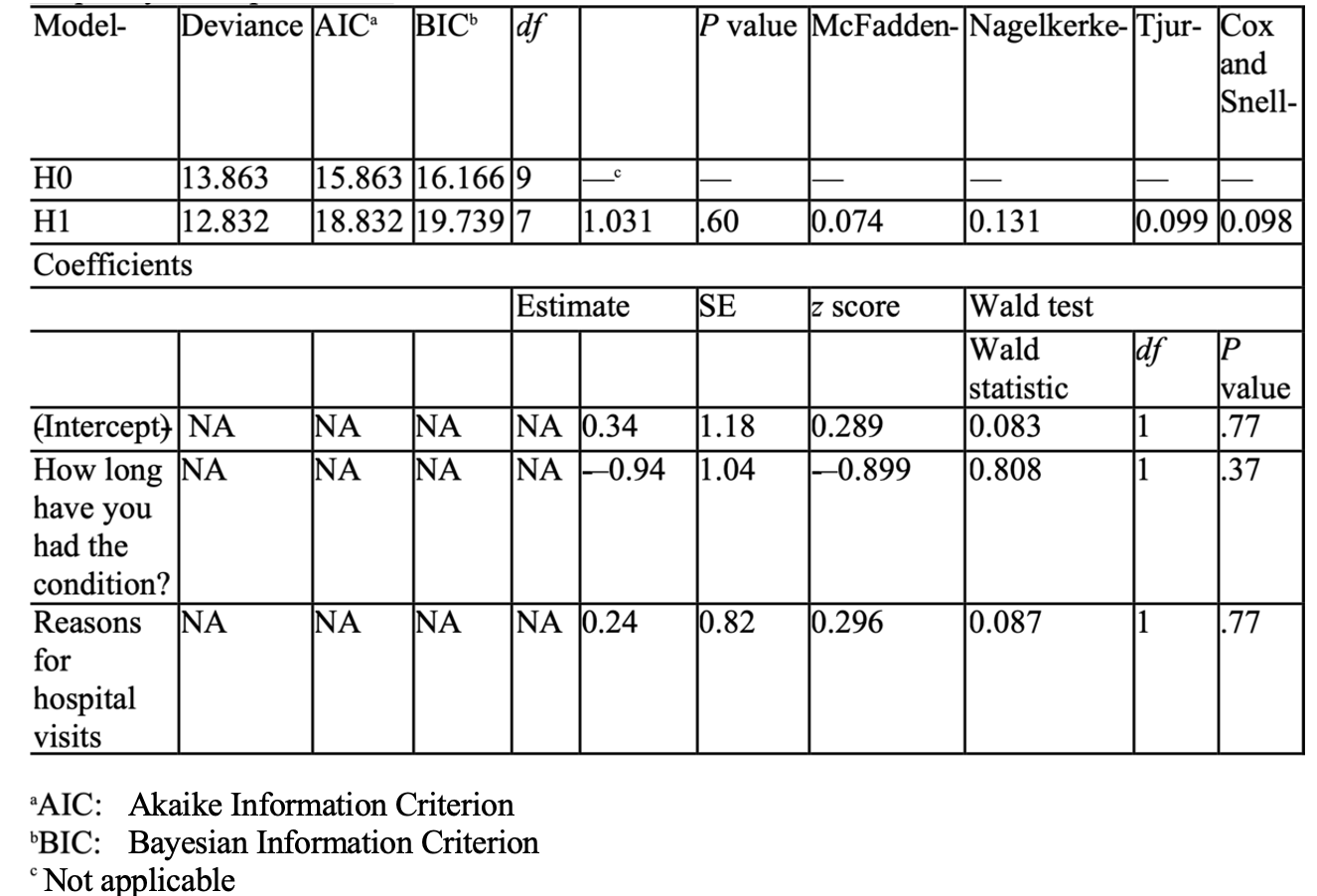

Supplement: Multimedia Appendix 1 [file diabetes_v9i1e56756_app1.png]

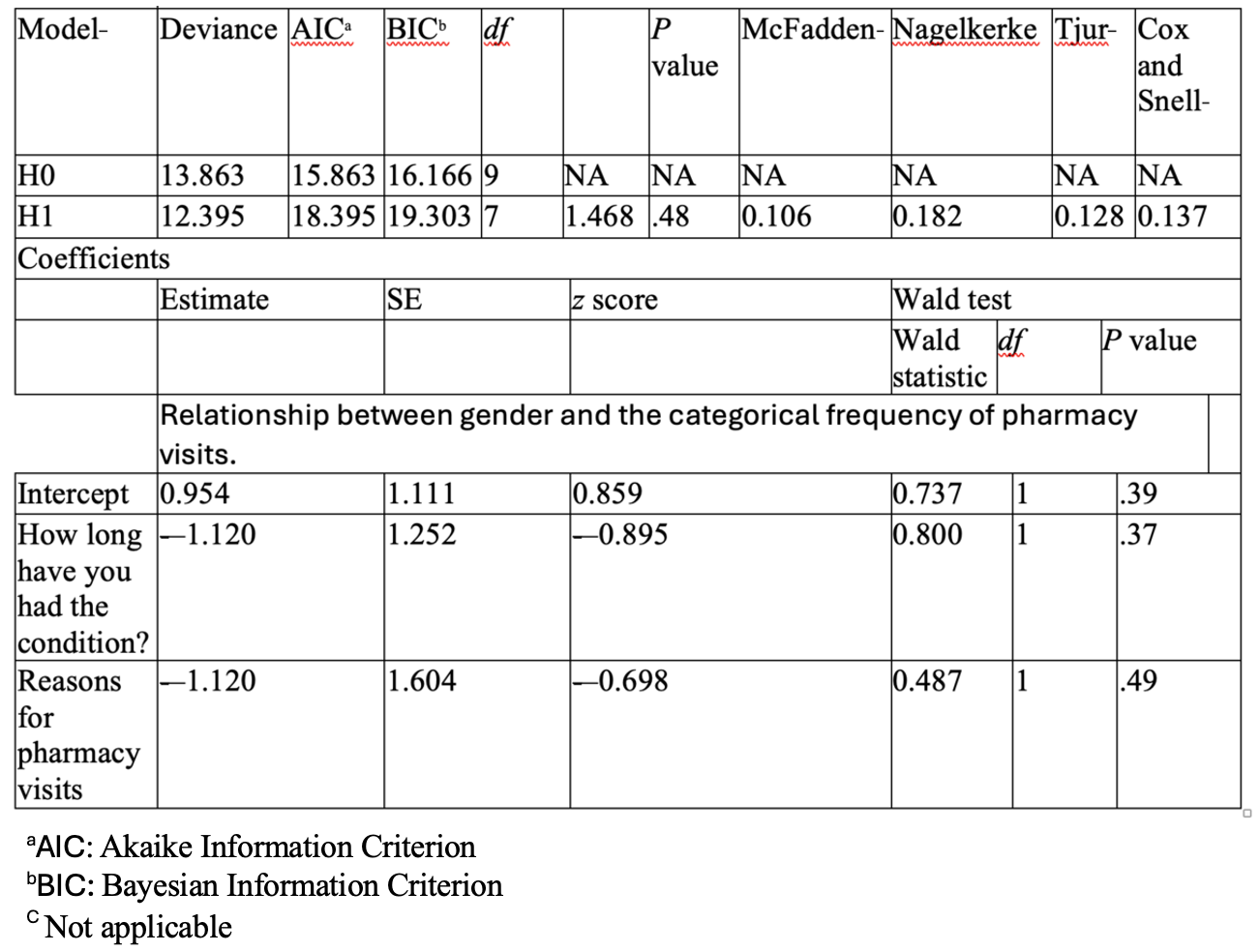

Supplement: Multimedia Appendix 2 [file diabetes_v9i1e56756_app2.png]
